# Landowner perceptions of the value of natural forest and natural grassland in a mosaic ecosystem in southern Brazil

**DOI:** 10.1007/s11625-015-0319-3

**Published:** 2015-07-07

**Authors:** Kirsten A. Henderson, Mateus Reis, Carolina C. Blanco, Valério D. Pillar, Rodrigo C. Printes, Chris T. Bauch, Madhur Anand

**Affiliations:** 1 School of Environmental Sciences, University of Guelph, Guelph, ON Canada; 2 Universidade Estadual do Rio Grande do Sul, São Francisco de Paula, Rio Grande do Sul Brazil; 3 Departmento de Ecologia, Universidade Federal do Rio Grande do Sul, Porto Alegre, Rio Grande do Sul Brazil; 4 Department of Applied Mathematics, University of Waterloo, Waterloo, ON Canada

**Keywords:** Restoration, Perception, Land use change, Forest–grassland mosaic, Policy

## Abstract

The forest–grassland mosaics of southern Brazil have been subject to many land use and policy changes over the decades. Like many grasslands around the world, the *Campos* grasslands are declining with few conservation efforts underway. In contrast, forests receive much attention and many incentives. It is hypothesized that perception of land cover has the potential to shape ecosystems. Here we conduct a questionnaire to further our understanding of decision-making practices that alter landscapes (*Campos* grassland, *Araucaria* forest, agriculture and plantation) and direct land policies in the region. Our analysis reveals that plantations are significantly less desirable than the other landscape types. However, plantation land use has increased by 87 % over the past few decades, as a result of industry and government incentives. The proportions of other landscape types have remained consistent over the past two decades. Restoration of native vegetation is not a priority of landowners and restoration would require a financial incentive.

**Electronic supplementary material:**

The online version of this article (doi:10.1007/s11625-015-0319-3) contains supplementary material, which is available to authorized users.

## Introduction

Deforestation has become notorious in tropical and subtropical South America due to the impact on biodiversity and the rapid rate of clearance (Laurance et al. 2001; Mähler-Júnior and Larocca 2009; Rodrigues et al. 2009). As a result, government and private programs have established numerous conservation initiatives. Pristine grasslands are also declining, yet with little in the way of conservation efforts (Bond and Parr 2010; Overbeck et al. 2007). These grasslands are sometimes erroneously considered to be degraded lands, a result of anthropogenic activities or the early successional stages of forests, while forests are perceived as more productive, pristine landscapes full of diversity (Parr et al. 2014). Likewise, land conservation in Brazil reflects a high forest bias (Soares-Filho et al. 2014).

The *Campos* grasslands form a mosaic with mixed forests on slopes and valleys in the east of the South Brazilian Plateau and *Araucaria* forest in the highlands of southern Brazil (Müller et al. 2012). The grasslands are considered the older vegetation type; however, recent wet climatic conditions have provided a favourable environment for forest development (Behling and Pillar 2007). Fire and grazing are the main disturbances that impede forest expansion (Overbeck et al. 2007; Müller et al. 2012). Other anthropogenic influences such as afforestation of grasslands with pine plantations, logging of *Araucaria* *angustifolia* forests and large-scale conversion of native vegetation for agricultural purposes have altered the landscape (Behling and Pillar 2007). It is estimated that 50 % of the Brazilian *Campos* is still natural grassland in Rio Grande do Sul (Cordeiro and Hasenack 2009) and only 12–16 % of the original landscape cover remains in the Atlantic Forest (Ribeiro et al. 2009).

Forest conservation takes precedence, following a history of forest degradation in the Atlantic Forest. The *Araucaria* forests have been extensively logged or cleared for agriculture, yielding a critical conservation status (FAO 2009). *A. angustifolia* is one of few indigenous gymnosperm tree species remaining in Brazil (Rizzini 1997), with important socioeconomic and ecological benefits (Auler et al. 2002). The number of non-native species plantations (pine and eucalypts) increased by 23 % across Brazil from 2005 to 2010 (ABRAF 2011). In terms of overall planted forest area, native forest species formed less than 5 % of planted areas, while pine and eucalypt species comprised 93 % of planted forests (MMA 2013). The state of Rio Grande do Sul recorded 14.4 % increase in non-native planted forests from 2005 to 2006 (MMA 2007). Government and social movements that promote regional development through agroforestry and, more likely, development of the forestry sector are thought to be responsible for the increase in planted area (Castro et al. 2008). Policy-makers often promote afforestation of grassland to increase productivity and mitigate elevated atmospheric carbon dioxide (Bond and Parr 2010).

Meanwhile, the native *Campos* grasslands of Brazil have undergone extensive land transformation. In the past three decades, approximately 25 % of the Brazilian *Campos* have been converted to agriculture or plantation forestry (Overbeck et al. 2007). In the 1990s, agricultural practices gained popularity with rice and soya crops occupying fertile soil and forest plantations composed of eucalypts and pine on poorer growing soils (Cordeiro and Hasenack 2009; Pillar et al. 2009). These grasslands represent important biodiversity sites and provide numerous ecosystem functions, in addition to supporting livestock production and the Gaucho culture (Lang 2013; Overbeck et al. 2013); however, only 0.15 % is formally protected as conservation units (Develey et al. 2008).

The Brazilian Forest Code (BFC) is responsible for the conservation of native vegetation on private lands (BFC 2012; Sparovek et al. 2012). The BFC was created in 1965 and enforced by the “environmental crimes law” established in the late 1990s (Environmental Crimes Act 1998), promoting the restoration and conservation of native vegetation (Gautreau and Vélez 2011; Soares-Filho et al. 2014). In 1965, the BFC imposed regulations and penalties, in effort to regulate land use on private properties, under the notion that forest is a common good (Stickler et al. 2013). In 1981, the Brazilian National Environmental Policy Law addressed the need to restore all degraded lands (MMA 1981). It follows that the requirements for conservation of native vegetation should constitute 80 % of property in the Legal Amazon (BFC 1989), 35 % in the *Cerrado* biome (savannas) and 20 % in other biomes (BFC 1965). However, agribusiness has persistently lobbied against the BFC, claiming the provisions of the bill directly impede agricultural production (Sparovek et al. 2012; Soares-Filho et al. 2014). After record high deforestation rates in 1995, predominantly as a result of agricultural sprawl, regulation changes were prompted, enforcing stricter laws on rural property land use. Revisions of the BFC in 2012 were elicited by agribusiness’ condemnation of the strict conservation laws. The most significant changes to the law involve amendments to lessen the regulations on watershed protection and riparian vegetation, as well as the elimination of hilltop native vegetation protection (BFC 2012; Novaes and Souza 2013). Furthermore, under the new regulations, land that was illegally altered prior to 2008 is exonerated from restoration requirements and property size-specific regulations were included, relaxing requirements on small properties.

On the state level, the government has been promoting alternative land use practices, primarily through increased tree cover. Silvicultural activities started in the late 1980s in Uruguay, Argentina and Rio Grande do Sul in Brazil, initiating competition among the countries to attract world corporate leaders from the cellulose industry (Gautreau and Vélez 2011). As a result, landowners received massive public subsidies. The 1992 State Forest Code forbid the use of fire to maintain grasslands (RS 1992), contributing to increased silvicultural activities (Bristot 2001; Teixeira 2011). In 2004, state environmental administration implemented Environmental Zoning for Silviculture Activity (ZAS) to regulate tree farms in *Campos* grasslands, according to ecological vulnerabilities defined at different spatial unit scales (Gautreau and Vélez 2011). The original ZAS regulations (2006) permitted 25–50 % of property to be planted with tree farms; larger properties were permitted to convert lower percentages of land to plantation. The latest version of ZAS regulations establishes a maximum percentage of plantation per mixed spatial unit (watersheds divided by landscape unit), a minimum distance between plantations and offers watershed protection. Moreover, Brazil’s environmental legislation requires each plantation of exotic trees to have an environmental permit. In addition, the Low-Carbon Agriculture program provides loans to increase agriculture productivity while reducing associated carbon emissions and supporting forest restoration (Soares-Filho et al. 2014). The native *Araucaria* forests in Brazil have further regulations prohibiting deforestation and promoting conservation. In 1965, the Brazilian Government banned the harvest of native vegetation in permanent preservation areas, which includes *A.* *angustifolia* (BFC 1965). As of 2001, the tree has been listed as critically endangered and export of the species has been prohibited (Thomas 2013).

Although the law requires all types of native vegetation to be protected, landowners are often unaware or do not acknowledge this (Overbeck et al. 2013). Throughout the years, the BFC and other state initiatives have experienced many changes to the legal requirements for preservation of native vegetation, known as Legal Reserves (LR), in addition there have been various incentive programs and conservation initiatives, resulting in non-conformity among landowners, while making it difficult to monitor the land use transitions (Stickler et al. 2013). Non-compliance has been an issue for policy-makers, particularly in the Amazon and Atlantic Forest biomes, over the past decades (Soares-Filho et al. 2014). A significant reason for non-compliance with LR standards is the substantial cost to landowners, not only from restoration efforts, but also through foregone income from crops and cattle ranching (Sparovek et al. 2012; Stickler et al. 2013).

Human behaviour has a significant influence on land use and ecosystem stability (Innes et al. 2013). Many studies have looked at the decision-making behind conservation ecology. The scarcity hypothesis refers to conservation based on economic returns from ecosystem resources, such as timber. When a resource becomes scarce, the market value increases, providing an incentive for individuals to protect the resource (Farber et al. 2002; Satake and Rudel 2007; Barbier 2013). Alternatively, the ecosystem service hypothesis states that environmental degradation associated with native vegetation loss increases the vulnerability of the system to further degradation, which reduces the incentive to restore ecosystem structure (Satake and Rudel 2007). Moreover, individuals frequently demonstrate imitation behaviour, following the decisions of media or respected individuals in a community (Moser 2010; Bauch and Bhattacharyya 2012). Moser (2010) underlines the importance of social norms when communicating about climate change; climate change initiatives are more likely to be accepted when the views conform to those of the social group and therefore, individuals who engage in conservation initiatives are unlikely to be ostracized.

Most land use research in Brazil has focused on the rates of decline and forest area lost, particularly in the Legal Amazon, and the role of policy-makers. Few studies have been conducted in the southern region of Brazil and even less is known about the forces driving land use transformation. The South and Southeast regions of Brazil are more heavily occupied and the majority of native vegetation is on private land (Sparovek et al. 2011). Approximately, 80 % of the Atlantic Forest biome is on private property (SOS Mata Atlântica 2012); hence the importance of enhancing our knowledge of the decision-making practices of landowners. The decisions landowners make and the resulting land changes form complex patterns in ecosystem landscapes. The main objective of our study is to gain knowledge on individuals’ preference and perception of conservation values (including sustainable use) of natural ecosystems, relative to their abundance in the region, and how this perception changes considering past and possible future landscape compositions in the forest–grassland mosaics of southern Brazil. It is hypothesized that individuals show no preference for one or the other native landscape type (*Araucaria* forest or *Campos* grassland), compared to non-native ones (agricultural land and non-native plantation). In addition, we hypothesize that decisions regarding land use and conservation are consistent with imitation and rarity-based behaviour.

## Methods

### Study area

The study area is located in the region of São Francisco de Paula (town: 29°27′03″S, 50°35′41″W at an altitude of 912 m), in the South Brazilian plateau, northeastern Rio Grande do Sul (RS), Brazil (*Planalto das Araucárias*). The regional climate is described as subtropical, with moderate temperatures (annual mean 14.5 °C) and high annual precipitation (mean 2252 mm) without a marked dry season (Silva and Anand 2011). The northeastern region of RS represents the southern limit of the Atlantic Forest distribution (Silva et al. 2009) and is characterized by forest–grassland mosaics composed of native *Campos* grassland and native *A. angustifolia*. The soil characteristics (Andosols or Umbrisols) are uniform through the forest and grassland landscape types (Silva and Anand 2011). The primary anthropogenic land uses are cattle grazing, logging practices, agriculture and tree plantations. The 300 km^2^ region around São Francisco de Paula is composed of 30 % native *Campos* grassland, 25 % native *Araucaria* forest and 45 % other human land use (agriculture and/or tree plantation) (Lang 2013).

### Sampling and analysis of land use

**Fig. 1:**
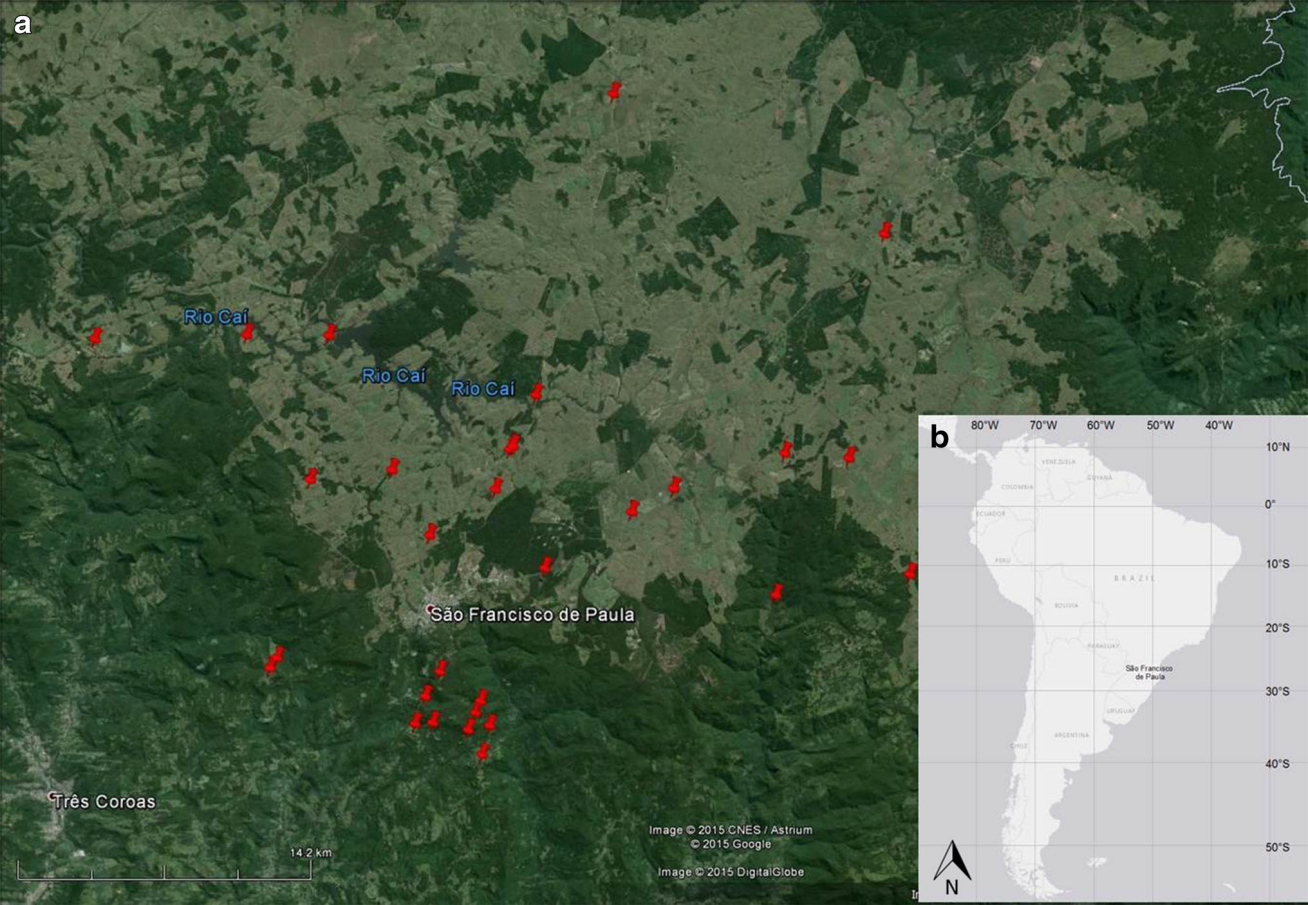
Location of the study site. **a** Map showing the 30 properties surveyed in the São Francisco de Paula region taken from Google Earth. **b** Inset of South America using ArcMap (ESRI 2012)

Data regarding the perception of changes and individual interactions with the natural environment were collected using a questionnaire. Thirty properties were selected using a stratified sampling technique, in which we chose to interview cattle ranchers with significant proportions of native vegetation on their land as our reference group. There was also an element of “snowball” sampling, where each farmer suggested the next person to survey. Figure 1 represents a site map of the properties surveyed. Each property was given a questionnaire (see Supplementary Information for full questionnaire) with ten questions concerning the past/present composition and preference for four landscape types (native *Campos* grassland, native *Araucaria* forest, agricultural land and non-native plantation), how experiences are shared with other people about land use and opinions about the importance of the conservation of native (i.e. unplanted) ecosystems. All composition values were gathered as a percentage of the landowner’s property. To assure landowners understood the classification of landscapes, landowners were shown images of native *Araucaria*, forest–grassland mosaic and native grassland prior to answering survey questions.

Questions 1 and 2 of the questionnaire relate to the composition of each landscape type on each landowner’s property and their preferred composition. Both current land composition/preference and land composition/preference from more than 10 years ago were considered to ascertain land use and perception changes in recent years.

The next set of questions aims to determine landowner decision-making practices and primary influences. With this we include changes in the landscape in a 5 km radius from the landowner’s property to explore whether imitation behaviour is present or if the scarcity hypothesis applies.

Questions 6 and 7 relate to the land composition in the São Francisco de Paula region. The purpose of these questions is to note the feedbacks between landowners’ preference and the composition of the region.

The final set of questions was devoted to gauging the interest of landowners to restore native *Araucaria* forest or native *Campos* grassland. Question 9 presents a hypothetical scenario in which landowners’ properties are composed entirely of *Araucaria* forest or *Campos* grassland; landowners were then asked how much of each native vegetation they would replace with the other. In addition, the questions explore the preference of restoring one native ecosystem over the other and the financial incentive required to convert anthropogenic land uses on their property to native vegetation.

Statistical analyses were performed using descriptive statistics and non-parametric tests (Wilcoxon signed rank test, Spearman correlation) in R (R Core Team 2012). Differences were considered statistically significant when *P* < 0.05.

## Results

### Composition and Preference

Relating to Q1, the native *Araucaria* forest comprises the greatest percentage of land use on surveyed properties, with an average of 35 %. Native *Campos* grassland makes up 30 % of the surveyed area, giving a combined native vegetation composition of 65 % with no significant difference between the amount of the two landscape types (*P* = 0.8642). The mean compositions of agriculture and non-native plantation land use are 20 % and 15 %, respectively. There is significantly less plantation land use (*P* = 0.01401) and agriculture land (*P* = 0.04075) compared to native *Araucaria* forest.

The preference for each landscape type (Q2 from questionnaire) does not vary significantly compared to the current composition landscape, with the exception of preference for more agricultural land use (*P* = 0.01417). On average, the mean preference for agriculture is 35 %, which is 15 % more agricultural land use than the current composition. The main reason landowners state for preferring agricultural land use is that agricultural practices are more profitable. The landscape composition preference for tree plantation is significantly less than the preference for both native ecosystems and agricultural land (*P* < 0.005). The landscape composition preferences for *Araucaria* forest, *Campos* grassland and agriculture do not vary significant from one another (*P* > 0.05).

### Composition and preference change

In general, composition on landowner properties has not changed significantly over the past three decades (Q3a from questionnaire). There has been a greater shift towards forested land, the mean tree plantation composition increased 87 %, the percent composition of plantation land use on properties increased from 8 to 15 % (*P* = 0.01418), while the mean percent composition of native *Araucaria* forest increased from 30 to 35 %, a 16 % increase in *A. angustifolia* composition over the years (*P* = 0.3244). The composition of *Campos* grassland decreased by 17 %, past mean compositions on individual properties decreased from 36 to 30 % (*P* = 0.05130). Likewise, the percentage of agriculture land use on individual properties decreased on average by 20 % from 25 to 20 %, (*P* = 0.8077). This decrease in grassland was perceived by 83 % of landowners within a 5 km radius of their property (Q5 from questionnaire). Within the 5 km radius surrounding their property, 52 % of landowners stated they observed a shift towards increasing *Araucaria* forest, while 34 % of landowners claim to have observed no landscape transitioning and 14 % of landowners stated they observed a shift towards decreasing forest. The correlation between the composition change of both native vegetation and the observed/perceived change in the 5 km radius is not significant for *Araucaria* forest (*ρ* = −0.001880, *P* = 0.9923), nor for *Campos* grassland (*ρ* = 0.1805, *P* = 0.3487). The majority of landscape changes occurred between 10 and 15 years ago.

Corresponding to Q3b, landscape composition preference has not changed significantly over the years. All *P* values for the change in composition preference are greater than 0.5. Few individuals changed their preference for *Araucaria* forest composition (*n* = 5) over the past decades; however, there exists a weak negative correlation between the slight increase in preferred composition and the perceived change in forest cover within the surrounding 5 km radius (*ρ* = −0.2602, *P* = 0.1729). Three landowners changed their preferred composition of *Campos* grassland; the minimal decrease in individual preference change for *Campos* grassland shows a weak positive correlation with the perceived decrease of grassland in the surrounding area (*ρ* = 0.2166, *P* = 0.2592). The negative correlation between the change in preferred forest composition and perceived forest cover change demonstrates rarity-based decision-making practices, inconsistent with the high discount rates on timber in Brazil (FAO 1998). The past landscape composition preference appears to be reflected in the current composition of land, which is positively correlated with the past preference for each landscape composition (Table 1).

**Table 1:** Correlation between past landscape composition preference and current composition using the Spearman correlation test

Landscape type	*ρ*	*P* value	Strength
Native *Campos* grassland	0.7454	0.000002289	Strong
Native *Araucaria* forest	0.6159	0.0002908	Strong
Plantation	0.3618	0.0248944	Moderate
Agriculture	0.3245	0.08024	Moderate

### Land use influence

The results from Q4 show that landowners are much more likely to gather information from someone they know with the same vocation (70 % of landowners, *P* = 0.02932), such as parents, neighbours or cooperatives. The other 30 % of landowners receive information from television, the newspaper or the internet. On a scale of 1–5, the average self-reported influences from both personal contacts and media are 3.3 and 3.4, respectively.

### Regional composition

Relating to Q6, landowners are unlikely to change their preference for *Araucaria* forest (*P* = 0.3938) or *Campos* grassland (*P* = 0.4313) after being informed of the current composition of land in the region. On average, the landowners would prefer to see the region of São Francisco de Paula composed of 39 % native *Campos* grassland, 29 % native *Araucaria* forest, 26 % agriculture and 2 % plantation (Q7 from questionnaire). The majority of landowners would prefer that non-native tree plantations be replaced by one of the three other landscape types (*P* < 0.00005) and would like to increase the income generated, in addition to the productivity of properties. Seventy-three percent (*n* = 22) of landowners stated a preference for agriculture and ranching in the region for financial reasons or food productivity. However, 17 % of landowners (*n* = 5) stated the importance of landscape services provided by *Araucaria* forests.

The preferences for *Araucaria* forest and tree plantation on individual properties show weak negative correlation with the preferences in the region (*ρ* = −0.04, *P* = 0.8335 and *ρ* = −0.3187, *P* = 0.08609, respectively). The preference for agriculture shows a moderate positive correlation with regional preference (*ρ* = 0.5088, *P* < 0.005) and grassland is strongly positively correlated to the regional preference (*ρ* = 0.6444, *P* < 0.0005).

### Restoration

Ninety-three percent of landowners (*n* = 28) are willing to restore *Araucaria* forest at the expense of *Campos* grassland (Q9 from questionnaire), versus an almost equal 90 % (*n* = 27) willing to restore grassland at the expense of forest (*P* = 1). Landowners consider on average transitioning 36 % of hypothetical full forest cover on their property to *Campos* grassland, while landowners consider converting 32 % of hypothetical full *Campos* cover to *Araucaria* forest (*P* = 0.5451). However, given the choice of restoring either *Campos* grassland or *Araucaria* forest on their current property (Q8 from questionnaire), the majority of landowners (67 %, *P* = 0.02415) would rather restore *Campos* grassland than *Araucaria* forest. There is no strong negative correlation between the desire to restore the native grassland vegetation and the observed/perceived decrease in *Campos* grassland (*ρ* = −0.09485, *P* = 0.6245). Two out of 30 landowners did not select either native landscape types for restoration.

**Fig. 2:**
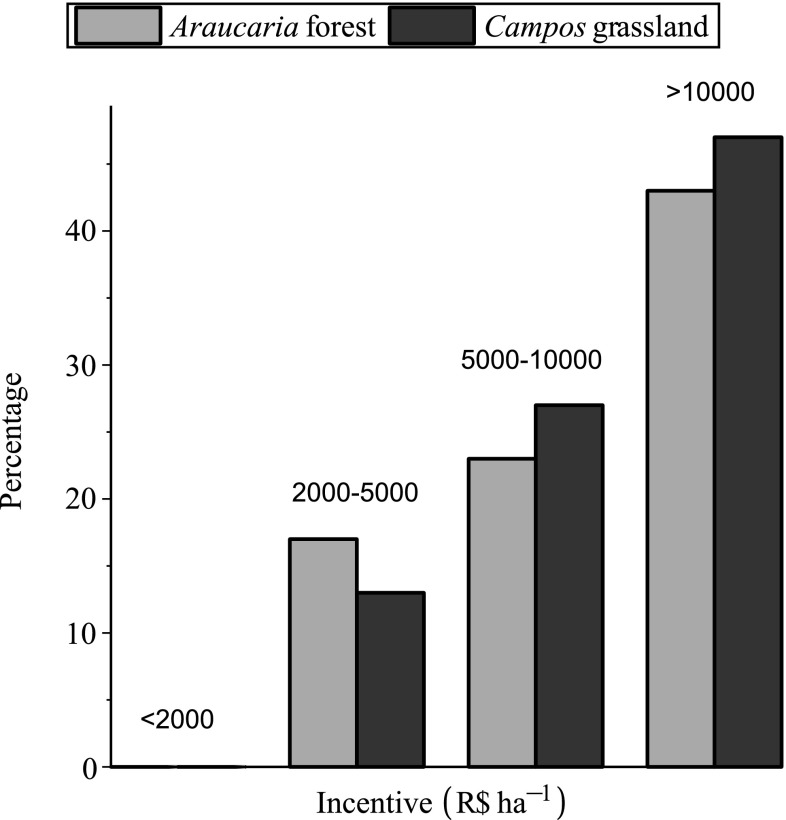
Required incentive to convert cropland to native vegetation. Q10 asks landowners how many Reals (R$) per hectare are required to consider converting cropland to native vegetation on their property. No landowners would restore native vegetation on their property without an incentive. Forty percent of landowners would restore both native vegetation landscapes for R$2000 (approx. US$765) to R$10000 (approx. US$3825) per hectare converted. The greatest proportion of landowners require more than R$10000 (approx. US$3825) per hectare converted to establish native vegetation (46 % for grassland, 43 % for forest). Some landowners would not restore native vegetation on their property for any amount of money (13 % for grassland, 17 % for forest)

The results from Q10 show that compensation for converted croplands does not vary significantly between *Araucaria* forest and *Campos* grassland (Fig. 2).

## Discussion

In past decades, restoration ecology has gained popularity in Brazil (Kageyama et al. 2003). Restoration ecology presents many challenges from environmental, social and economic perspectives. Our study offers insight into managing land use and behaviour that contribute to ecosystem transformations.

The literature indicates that landowners tend to prefer agricultural land use (Overbeck et al. 2007) and grasslands for their ability to support livestock production (Overbeck et al. 2013). Furthermore, the current value of one hectare of grassland is approximately five times greater than the same area of forest (R. Printes, personal communication). Despite the vast majority of the written comments stating a preference for native *Campos* grassland and agriculture for their ability to generate greater profits and productivity, *Araucaria* forest comprises the highest average composition in the region and there has been a decrease in *Campos* grassland over the past decades. This could be a result of the perceived benefit from forest ecosystem services or the laws that promote forest conservation and prohibit logging of the native *Araucaria* forest. Seventeen percent of landowners stated ecological services as a reason for maintaining forest, as well as the attraction of tourists. From landowner responses, we deduce that ecosystem services influence land management practices, in agreement with the ecosystem service hypothesis.

Forest transition in Brazil generally results from urbanization, industrialization, conservation, agricultural demand, rural exodus or land value changes (Baptista 2008). Many studies have described forest encroachment into adjacent grasslands (Asner et al. 2004; Behling and Pillar 2007; Silva and Anand 2011; Müller et al. 2012). Forest encroachment provides a well-founded explanation for the decrease in grassland and the increase in both native and exotic tree species in the region. Seed dispersal and germination are very effective at expanding over nearby grasslands (Bustamante and Simonetti 2005), especially as the climate becomes warmer and moister with increased carbon dioxide in the atmosphere. The change in legislation that prohibits the logging and export of *A. angustifolia* and encourages conservation appears to have slightly increased the native forest cover in the region. Furthermore, it has been suggested that fire suppression in the region is an important contributor to the expansion of forest cover (Behling and Pillar 2007).

Plantations were the only landscape type to experience a significant increase in the last 30 years. Lang (2013) noted an increase of 92.4 % in plantation area, consisting mostly of *Pinus* sp. in the *Campos de Cima da Serra* region (subtropical highland grasslands, northeastern Rio Grande do Sul). We suggest that the government and private industry incentives for the cultivation of exotic species prompted an increase of silvicultural activities in the area. In addition, the forestry industry strongly influences the bans placed on fire (R. Printes, personal communication), favouring the development of plantation trees. There has been a marked increase in recent years of plantation land use, contrary to the average landowner’s preference to reduce the composition of tree plantation on their property. Plantation land use is significantly less preferred than any other landscape type, possibly as a result of the high discount rates on timber in Brazil, which can make monoculture plantations economically unviable when considering production costs and profits (Oliveira et al. 1998; Soares et al. 2003).

The decrease in agricultural land use could be evidence of intensified law enforcement over the years noted by Soares-Filho et al. (2014) or marginal growing conditions on the slopes south of São Francisco de Paula, causing landowners to abandon agriculture practices. Historically, agribusiness has taken advantage of weak enforcement and ineffective monitoring (Nepstad et al. 2014). When discussing the reasons for changing their land use, most of the landowners made changes that generated greater profits, while some landowners remarked a change in legislation that prohibited logging and certain management strategies. Grazing and fire as maintenance strategies for grasslands are not approved by the Brazilian conservation policy in protected areas (Overbeck et al. 2013), though fire use for land management is allowed on the majority of grasslands with permission from the municipal government (RS 2012). For the small percentage of grassland in protected areas, the ban on grazing and fire could contribute to the observed decrease in *Campos* grasslands (Oliveira and Pillar 2004).

Numerous studies have demonstrated the impact of human behaviour on landscape transitions either directly through preference for a given vegetation type and management strategies or indirectly through climate change (Bennett and Willis 2000; Asner et al. 2004; Horan et al. 2011; Innes et al. 2013). The amendments to the BFC and other conservation initiatives at the state level for Rio Grande do Sul provide some insight into the shifts in resource and ecological services perceptions over the decades. This study aims to determine what drives decision-making. The questionnaire shows that landowners are much more likely to seek information from individuals with similar experiences. The perceived change in composition within a 5 km radius surrounding the properties slightly influences the preference for the landscape type; however, the change in native vegetation composition on individual properties does not appear to be affected by the changes in the region. Human decision-making follows a set of valuation processes, in which individuals often select the outcome with the greatest perceived utility, the most immediate return and the greatest degree of certainty (Rangel et al. 2008; Moser 2010). Our results are consistent with decision-making practices described by Rangel et al. (2008) and Moser (2010).

From our results, it is not possible to make any conclusive remarks on the use of rarity-based decision-making practices in the region; we argue that the region has not experienced significant enough declines in native vegetation cover to generate concern that would be reflected in the questionnaire. The high discount rates in Brazil decrease gains from timber and often preclude scarcity conservation (FAO 1998; Oliveira et al. 1998). There is a significant difference in composition and preference for *Araucaria* forest compared with plantations (*P* = 0.001261), despite the greater profits from non-native plantations (Cubbage et al. 2007), which reflects landowner conservation values for native forests. The *Campos* grasslands are important to the gaucho culture and provide significant income to the region (Lang 2013; Overbeck et al. 2013), which is consistent with landowner comments. From landowner responses, we infer that landowners maintain land that generates the most profit while adhering to laws and regulations.

From the collected data, we gather that the preferred landscape on an individual property reflects the desired composition in the region and the information given about the region does not change the preference of landowner’s composition. We argue that landowners in the region have a good understanding of the land composition in their region and thus the information given about the area had no impact on their preference because they were already aware of the regional ecosystem composition. The landscape composition of the region is similar to the average and mode compositions on individual property, which may suggest imitation behaviour.

Rates of transformation and degradation are not uniform throughout Rio Grande do Sul (Cordeiro and Hasenack 2009). The majority of southern Rio Grande do Sul falls within 80–100 % compliance with the 2012 BFC (Soares-Filho et al. 2014). The region of our study showed an over 80 % compliance with the 2012 BFC and within this region landowners were shown to maintain between 0.8 and 3 times the required native vegetation. Similarly, our results indicate that the composition on each individual property varies widely, while 93 % of landowners maintained at least 20 % combined native vegetation. Considering the state of RS has a surplus of 664,000 ha of land in the Atlantic Forest biome, with the potential to be converted from native vegetation to other land uses and 3 million surplus ha of land in the Pampa biome (Soares-Filho et al. 2014), we reason that the 7 % of landowners with less than 20 % LR may legally be permitted to maintain a lower composition of native vegetation through the Environmental Reserve Quota; whereby, landowners can trade their excess required native vegetation quota with others, in the same biome, that failed to meet the LR (Soares-Filho et al. 2014).

One very clear pattern that emerged from the respondents is that restoration can be costly and large incentives are required by landowners to restore native vegetation at the expense of croplands. Strict regulations can result in compliance issues, as seen by increasing the LR requirement (Stickler et al. 2013). From the comments on the questionnaires it is evident that land productivity and profit are important to landowners, we argue more important than restoring native vegetation. Landowner responses concerning the amount required for restoration indicate that restoration is viewed as selling land to conservation authorities. The average cropland is valued at approximately R$9500 (approx. US$3625) (IFMB 2011), whereas forested land is valued at R$1000 (approx. US$380) and grassland at R$5000 (approx. US$1910) (R. Printes, personal communication). Our work suggests that landowners wish to profit from restoration initiatives or reclaim lost revenue from cropland conversion. The majority of landowners would rather restore native *Campos* grassland on their property at the expense of croplands since grasslands inherently support ranching practices, the sole income for many of the landowners. Conversely, landowners demonstrate equal willingness to restore both native ecosystems at the expense of the other in the hypothetical situation.

## Conclusion

This analysis provides useful information for policy-makers by quantifying changes in land management and preference of land use in São Francisco de Paula over the past decades. We must take into consideration that surveyed landowners maintain properties in the region with the highest composition of native vegetation and thus are likely to be in compliance with land management regulations. While the collection of landowner knowledge has some inherent bias it does not appear to include deliberate misinformation, the results are consistent with landscape changes noted in other studies of the region, as well as changes in policy (Oliveira and Pillar 2004; Overbeck et al. 2013; Soares-Filho et al. 2014). The major study results indicate that there is no preference for either of the native vegetation types, in agreement with our null hypothesis. In addition, we find that landowners have no significant preference for the proportion of landscape composed of agriculture over native vegetation, while plantations are clearly less desirable than any other landscape. From landowner written responses, we conclude that land productivity and profit are priorities for the majority of landowners. However, maintenance of ecosystem services also influences the decision-making of many landowners. Sampling of other landowners’ preferences further influences individuals, while the proportion of native vegetation does not seem to be low enough to test the scarcity hypothesis. Restoration of native vegetation is not a priority of landowners and any form of restoration would require a financial incentive. Our results suggest that the economics of restoration and conservation are crucial to the success of policies and the maintenance of native ecosystems.

The responses of landowners to the questionnaire give an indication of when changes in legislation have occurred and whether or not they were successful. Landowners in the region of São Francisco de Paula show a high compliance with regulations and this in turn appears to maintain some level of conservation of the native vegetation. Reflecting on past land management strategies and adapting future policies to include information on landowner preferences can circumvent unsuccessful strategies and promote positive changes (Fensham and Fairfax 2003). The data collected on land composition in the region enhance our understanding of land management strategies and vegetation composition for future fire regulation policies. The results will be used in future analysis to parameterize land use behavioural models.

## Electronic supplementary material

Below is the link to the electronic supplementary material.

Supplementary material 1 (DOCX 5060 kb)
