# Supplementary material for: Landowner perceptions of the value of natural forest and natural grassland in a mosaic ecosystem in southern Brazil
Source: Sustain Sci. 2015 Jul 7;11(2):321–30. doi: 10.1007/s11625-015-0319-3 (PMC6106652; doi:10.1007/s11625-015-0319-3)
Supplement: Supplementary file 1 — Supplementary material 1 (DOCX 5060 kb) [file 11625_2015_319_MOESM1_ESM.docx]

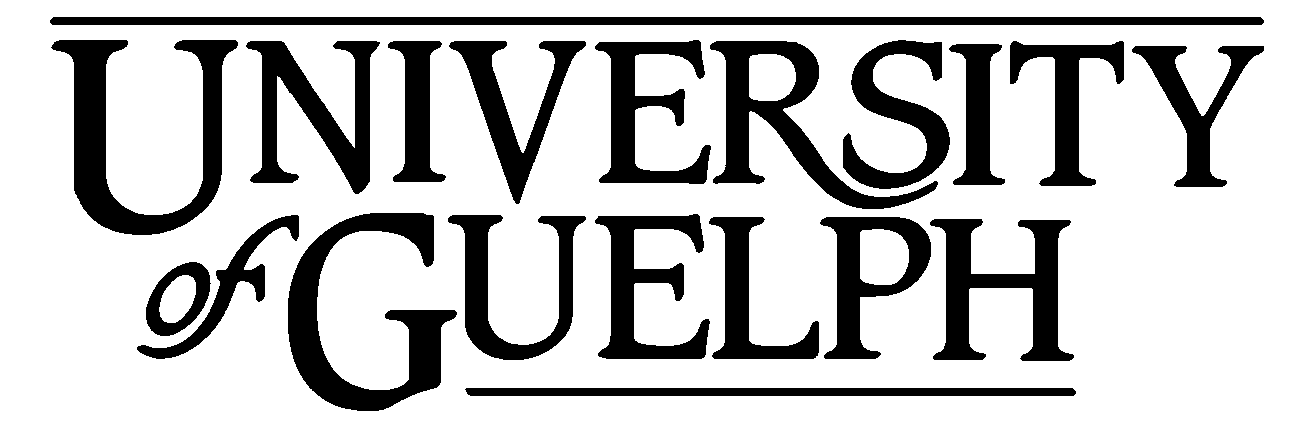


**SCHOOL OF ENVIRONMENTAL SCIENCES**

**UNIVERSITY OF GUELPH**

**GUELPH, ON, CANADA**

**- QUESTIONNAIRE FOR SOCIOLOGICAL SURVEY -**

**Project title: Coupled human-environment system dynamics and alternative stable states in complex mosaic ecosystems**

**Guide to the interviewer:**

This questionnaire is part of a research project under the coordination of the University of Guelph, Canadá, and in collaboration with the Universidade Estadual do Rio Grande do Sul, Brazil (UERGS). The cover letter attached to this questionnaire presents a more formal introduction and detailed information of the research project. The cover letter must be read to and discussed with each participant. A copy should be left with each participant, and proper explanations and clarifications should be given if required. If employees are being approached, care must be taken that there is no pressure to take part on part of the employer – nor should the employer receive any identified data. Bellow is some general information to guide you for the application of this questionnaire.

*Main objectives:* The main objective of this questionnaire is to know what is the people’s perception about conservation values (including sustainable use) of natural ecosystems, relative to their abundance in the region, and how this perception change considering possible past and future landscape compositions.

*Where to apply the questionnaire:* South Brazilian plateau in northeastern RS, Brazil (*Planalto das Araucárias)*. 30 properties should be selected among those with higher proportions of remaining areas of natural mosaics between *Campos* and *Araucaria* forest in the region of **São Francisco de Paula.**

*Target:* Interviews should be applied preferably to the landowner of the properties.

**Introduction:**

**(To be read/explained to the respondents, before starting the questionnaire)**

*Araucaria* forest (i.e., not planted) and *Campos* grasslands (not planted, but could still have grazing by cattle which we consider to be “natural”) are the two original native vegetation types in the region, and in some areas they have been coexisting side by side for centuries, forming mosaics in the landscape **(show Figure 1)**.

However, the occurrence of both ecosystems in the region has been reduced very fast over the last centuries, to account for the necessity of using the land for other purposes (e.g. agriculture, forestry, planted pastures, etc.). Therefore, we would like to know how you perceive these changes and interact with the natural environment, how you share your experiences with other people about land use, and what your opinion is about the importance of the conservation of native (i.e. unplanted) ecosystems. Ten questions will be asked in the following questionnaire. There are no right or wrong answers. We only want to know your opinions.

**Figure 1: Native vegetation types and their occurrence in the region (area of São Francisco de Paula).**

Q1. What is the current landscape composition your property?

[Interviewer should make sure these add up to 100%]

**% native *Campos* grassland: _________ %**

**% native *Araucaria* forest: _________ %**

**% agriculture: _________ %**

**% non-native tree plantation: _________ %**

**Please explain the land-use if there is agriculture or tree plantation (e.g., type of crops or tree species planted):**

|  |
| --- |
|  |

Q2. Which landscape composition do you prefer on your property (if different from Q1) and why?

[Interviewer should make sure these add up to 100%]

**% native *Campos* grassland : _________ %**

**% native *Araucaria* forest: _________ %**

**% agriculture: _________ %**

**% non-native tree plantation: _________ %**

**Why do you prefer this composition?**

**_______________________________________________________________________________________**

**_______________________________________________________________________________________**

**_______________________________________________________________________________________**

Q3. Have you been on your property for more than 10 years? If yes, was there a time in the past 10 years when you….

A) preferred a different landscape composition?  If so, what was it?

[Interviewer should make sure these add up to 100%]

**% native *Campos* grassland : _________ %**

**% native *Araucaria* forest: _________ %**

**% agriculture: _________ %**

**% non-native tree plantation: _________ %**

**Why do you prefer this composition?**

**_______________________________________________________________________________________**

**________________________________________________________________________________________**

**________________________________________________________________________________________**

**How many years ago did you change your mind?**

**_____________________________________________________________________________________**

B) actually had a different landscape composition?  If so, what was it?

**% native *Campos* grassland : _________ %**

**% native *Araucaria* forest: _________ %**

**% agriculture: _________ %**

**% non-native tree plantation: _________ %**

**Why do you have this different composition?**

**_______________________________________________________________________________________**

**________________________________________________________________________________________**

**How many years ago did you change your landscape composition?**

**_____________________________________________________________________________________**

| Q4. | | When deciding about land use on your property (e.g. change management or change land use activity), do you ask someone for advice (e.g., neighbor, parents, cooperative members, etc)? | | |
| --- | --- | --- | --- | --- |
|  | | | | |
|  | **Yes ->** | **With whom do you usually talk about it?** |  |  |
|  |  |  | |  |
|  |  | **What is his/her profession? __________________________** | |  |
|  |  | **In a scale of 1 to 5 (1 = not at all influential; 5 = very influential), what is the influence of this opinion in your decisions? _____________** | |  |
|  |  |  | |  |
|  | **No ->** | **What kind of other sources of shared information available most influence you on this decision (e.g. TV, radio, newspaper, internet, etc):______________** | |  |
|  |  |  |  |  |
|  |  |  | |  |
|  |  | **In a scale of 1 to 5 (1 = not at all influential; 5 = very influential), what is the influence of this information in your decisions? _____________** | |  |
|  |  |  |  |  |

| Q5. | What change have you been noticing or heard about in the 5km radius of your property? | | |
| --- | --- | --- | --- |
| [interviewer should circle their responses below]. | | |  |
| Land cover of native *Campos* grasslands **increased/decreased/stayed the same.** | | |  |
| Land cover of native *Araucaria* forests **increased/decreased/stayed the same.** | | |  |
|  | |  | |

Q6. According to recent studies, in this region (300 km^2^ around São Francisco de Paula) the landscape composition is:

% native *Campos* grassland (including areas grazed by cattle): 30%

% native *Araucaria* forest*:* 25%

% other human land use (agriculture and/or plantation) : 45%

Considering this information, does your preference indicated in Q2 change? If so, which landscape composition would you now prefer?

[Interviewer should make sure these add up to 100%]

**% native *Campos* grassland : _________ %**

**% native *Araucaria* forest: _________ %**

**% agriculture: _________ %**

**% non-native tree plantation: _________ %**

Why do you prefer this composition?

**_______________________________________________________________________________________**

**________________________________________________________________________________________**

**________________________________________________________________________________________**

Q7. What is your preferred landscape composition in the region (area of São Francisco de Paula)?

[Interviewer should make sure these add up to 100%]

**% native *Campos* grassland : _________ %**

**% native *Araucaria* forest: _________ %**

**% agriculture: _________ %**

**% non-native tree plantation: _________ %**

Why do you prefer this composition?

**__________________________________________________________________________________________**

**__________________________________________________________________________________________**

**__________________________________________________________________________________________**

Q8. Suppose you wanted to restore natural vegetation on your land, which would you prefer to restore?

|  | **Native *Araucaria* forest** |
| --- | --- |
|  | **Native *Campos* grasslands** |

Q9. Suppose on your property the only natural vegetation is native *Araucaria* forest. **If this were the case, do you think *Campos* grasslands should be restored on your property at the expense of native *Araucaria* forest*?* If yes, how much (in % relative cover)?**

_______________________________________________

Suppose on your property the only natural vegetation is native *Campos* grassland**. If this were the case, do you think *Araucaria* forest should be restored on your property at the expense of native *Campos* grassland*?* If yes, how much (in % relative cover)?**

_______________________________________________

[interviewer, please make sure they answer both of the questions above]

Q10. If you have/had a crop on your land (agricultural or tree plantation), how much would money per hectare would you accept to convert your cropland into

1. **native *Araucaria* forest? ____________________**

**If you don’t have a specific value in mind, please answer one of these:**

1. **< R$2000**
2. **R$2000-5000**
3. **R$5000-10.000**
4. **>R$ 10.000**
5. **native *Campos* grassland (could be with cattle)? ____________________**

**If you don’t have a specific value in mind, please answer one of these:**

1. **< R$2000**
2. **R$2000-5000**
3. **R$5000-10.000**
4. **>R$ 10.000**

Cover Letter

We thank you for agreeing to participate in this research. This research is being coordinated by the University of Guelph (School of Environmental Sciences), Canadá, and in collaboration with the Universidade Estadual do Rio Grande do Sul, Brazil.

The main objective of this research is to predict how the landscape in this region will be in the future, by the analysis of the interactions between the natural environment and human’s decisions about land use. For this, we would like to know how you perceive and interact with the natural environment, how you share your doubts, knowledge and experiences with other people about land use, and what is your opinion about the importance of the conservation of native (i.e. unplanted) ecosystems.

All these questions will be asked in the following questionnaire, and would take approximately 30 minutes. There are no right or wrong answers, we only want to know your opinions, and you can decline to answer any question(s). There will be no risks, discomforts and/or inconveniences to you (including physical, psychological, emotional, financial and social) on participating in this survey.

Your participation in this research is very important and the results can help you and the local community to plan long-term sustainable managements that guarantee community benefits by land use, in a way that preserves its natural state for future generations. Research findings will be available in the public library or local partner institution (UERGS library) closest to you.

All the information obtained with this questionnaire will be used only for research and teaching purposes, and we ensure the confidentiality of all your personal information to the best of our ability.

The project has been reviewed and received ethics clearance through the University of Guelph Research Ethics Board. If you have any questions regarding the use and safety of human subjects in this research project you may contact S. Auld, Director, Research Ethics, 519-824-4120, ext. 56606, [reb@uoguelph.ca](mailto:reb@uoguelph.ca).

Sincerely,

________________________________________

Prof. Madhur Anand, project coordinator

School of Environmental Sciences

University of Guelph

**Contact information:**

**Madhur Anand (principal investigator)**

School of Environmental Sciences, University of Guelph. 50 Stone Road East.

Edmund C. Bovey Building, Room 2241. Guelph, ON, Canada. N1G 2W1.

Phone: 519-824-4120 x56254

e-mail: [manand@uoguelph.ca](mailto:manand@uoguelph.ca)

**Chris Bauch (co-investigator)**

Department of Applied Mathematics, University of Waterloo. 200 University Avenue West Waterloo, ON, Canada. N2L 3G1.

Phone: 519-888-4567 ext. 32250

e-mail: [cbauch@uwaterloo.ca](mailto:cbauch@uwaterloo.ca)

**Rodrigo Cambará Printes (co-investigator)**

Laboratory of Environmental Management and Negotiation of Conflicts. Universidade Estadual do Rio Grande do Sul. Rua Assis Brasil, 842. São Francisco de Paula, Rio Grande do Sul, Brazil. CEP: 95400-970.

Phone: +55 54 99260351

e-mail: cambara7@gmail.com
